# The Interstitial System of the Brain in Health and Disease

**DOI:** 10.14336/AD.2020.0103

**Published:** 2020-02-01

**Authors:** Ashok K. Shetty, Gabriele Zanirati

**Affiliations:** ^1^Institute for Regenerative Medicine, Department of Molecular and Cellular Medicine, Texas A&M University College of Medicine, College Station, TX 77843, USA; ^2^Brain Institute of Rio Grande do Sul (BraIns), Pontifical Catholic University of Rio Grande do Sul (PUCRS), Porto Alegre, RS, Brazil

**Keywords:** beta-amyloid, cerebrospinal fluid, extracellular matrix, extracellular vesicles, glymphatic system, interstitial fluid, phosphorylated tau

## Abstract

The brain interstitial fluid (ISF) and the cerebrospinal fluid (CSF) cushion and support the brain cells. The ISF occupies the brain interstitial system (ISS), whereas the CSF fills the brain ventricles and the subarachnoid space. The brain ISS is an asymmetrical, tortuous, and exceptionally confined space between neural cells and the brain microvasculature. Recently, with a newly developed *in vivo* measuring technique, a series of discoveries have been made in the brain ISS and the drainage of ISF. The goal of this review is to confer recent advances in our understanding of the brain ISS, including its structure, function, and the various processes mediating or disrupting ISF drainage in physiological and pathological conditions. The brain ISF in the deep brain regions has recently been demonstrated to drain in a compartmentalized ISS instead of a highly connected system, together with the drainage of ISF into the cerebrospinal fluid (CSF) at the surface of the cerebral cortex and the transportation from CSF into cervical lymph nodes. Besides, accumulation of tau in the brain ISS in conditions such as Alzheimer’s disease and its link to the sleep-wake cycle and sleep deprivation, clearance of ISF in a deep sleep via increased CSF flow, novel approaches to remove beta-amyloid from the brain ISS, and obstruction to the ISF drainage in neurological conditions are deliberated. Moreover, the role of ISS in the passage of extracellular vesicles (EVs) released from neural cells and the rapid targeting of therapeutic EVs into neural cells in the entire brain following an intranasal administration, and the promise and limitations of ISS based drug delivery approaches are discussed

## Introduction

**Figure 1.**
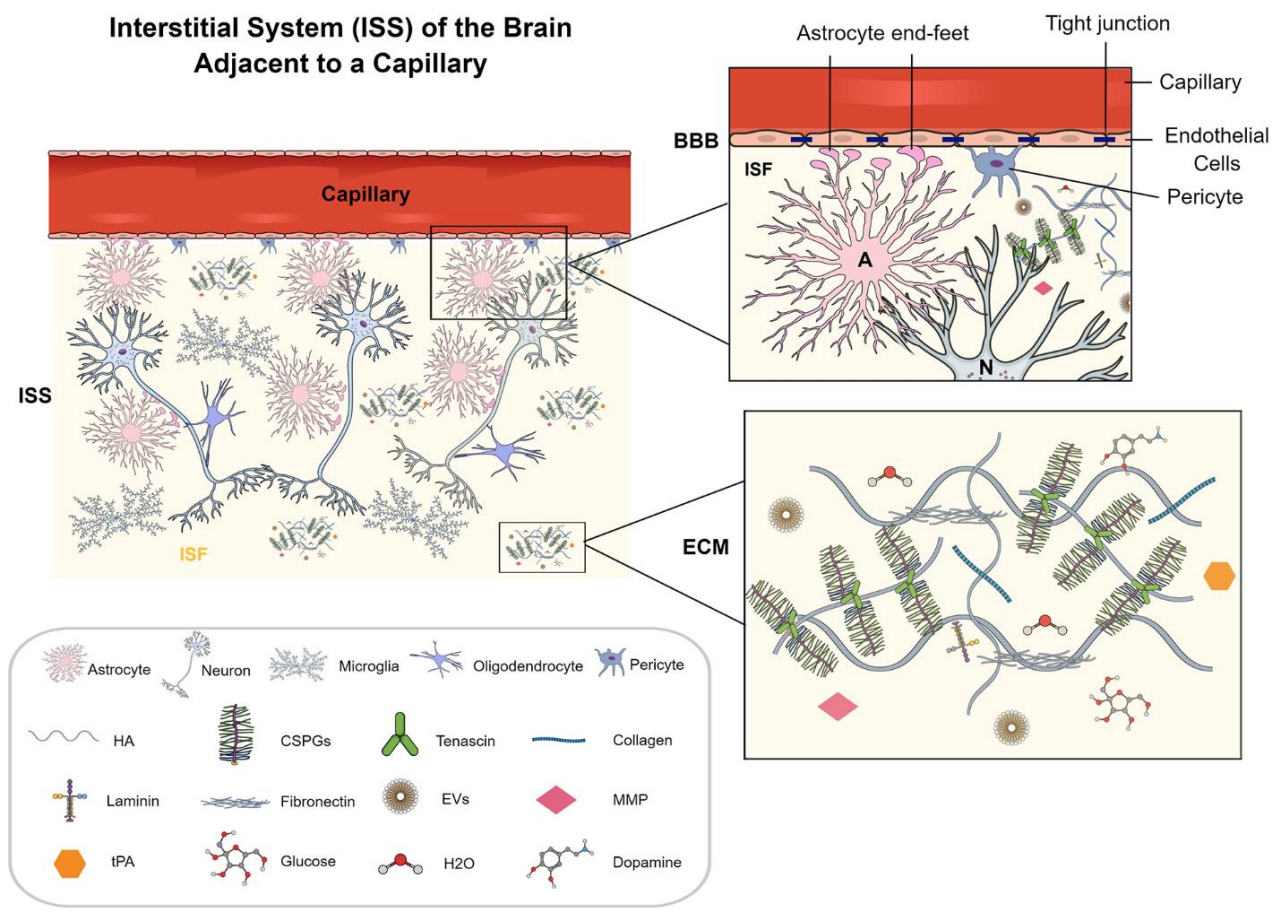
**A cartoon illustrating the brain interstitial system (ISS) between neural cells comprising interstitial fluid (ISF) and the extracellular matrix (ECM), adjacent to a brain microvasculature**. The magnified view of a portion of the cartoon on the top right shows endothelial cells with tight junctions, astrocyte end-feet, and pericytes at the interface of ISS and the microvasculature. The magnified view on the bottom right shows the ECM with its structure and components. The ECM contains hyaluronic acid, proteoglycans - CSPGs, tenascin, and a small amount of collagen, laminin, and fibronectin. Some components of ISF are represented, including EVs, H2O, glucose, dopamine, matrix metallopeptidase (MMP), and tissue plasminogen activators (tPAs). The illustrated ECM is distributed as the neural interstitial matrix in the space between neural cells. A, astrocyte; BBB, blood-brain barrier; N, neuron.

The brain is made up of neural cells such as neurons, astrocytes, oligodendrocytes, and microglia, the vasculature comprising arteries, arterioles, capillaries, venules and veins, and the interstitial system (ISS) The brain cells are cushioned and supported by two forms of brain-specific fluids, the brain interstitial fluid (ISF) and the cerebrospinal fluid (CSF) [1, 2] The ISF occupies the brain ISS, whereas the CSF fills the cerebral ventricles and the subarachnoid space [1, 2] The brain ISS is a dynamic and complex space connecting the vascular system and neural networks, which is composed of ISF and the extracellular matrix (ECM) (Fig 1) The ISS is the primary compartment of the brain microenvironment that provides the immediate accommodation space for neural cells, which accounts for 15-20% of the overall brain volume [1, 2] The space between the adjacent neurons, contiguous glia, or the adjoining neurons and glia is also known as the extracellular space (ECS) [1-3]

## The geometry of the brain ISS

The ISS is circumscribed by the plasma membrane of neurons or glia and the wall of blood vessels [4] (Fig. 1). However, the magnitude, locus, and proportions of the brain ISS are not static. Significant transformations ensue in ISS during the development of the brain due to events such as neurogenesis, neuronal migration and differentiation, expansion of dendritic tree, synapto-genesis, gliogenesis, synaptic stripping by microglia, and myelination [1, 5-10]. The geometry of the brain ISS also undergoes modification in aging due to the loss of synapses, neurons, dendritic regression, changes in morphology and numbers of glia, senoinflammation, and deposition of amyloid plaques [11-15]. In conditions such as epilepsy, the synchronized firing of a population of neurons may considerably change the geometry of ISS as well as the ISF drainage [16]. Such changes may also contribute to the maintenance of epilepsy because an accumulation of macromolecules within the brain parenchyma could alter the extracellular ionic equilibrium, and impact neuronal excitability [17].

## Composition of ISS

The constituents of the ISS comprise ISF, ECM, and diffusely distributed interstitial matrix (ISM) [1] (Fig. 1). The brain ISF, a water solvent containing ions, and gaseous and organic molecules such as proteins, peptides, enzymes, dopamine, and extracellular vesicles (EVs) secreted by cells, and the moving chains of glycoproteins attached to the ECM (Fig. 1). The ISF provides a direct medium for the supply of nutrients, removal of waste, and intercellular communication by encasing and incessantly soaking the neural cells [1, 18-20]. The source of ISF is still being investigated. Some studies have implied that ISF originates from the cerebrospinal fluid (CSF), cell metabolism, and the vascular system [21-24]. The exchange of substances between the ISS and the neural cells happens continuously [25]. A higher osmolarity in neural cells guides water molecules from the brain ISS into neural cells via aquaporin 4 (AQP4) and Na^+^/K^+^-ATPase enzymes in the cell membrane [26, 27].

The ECM, an abundantly hydrated net-like assembly produced by cells and disintegrated by enzymes, comprises collagen, elastin, glycosaminoglycans, proteoglycans, and glycoproteins such as tenascin, reelin, laminin, and fibronectin [1, 28-30] (Fig. 1). Besides, the ECM contains perineuronal nets (PNNs) and pericapillary matrix (PCM). PNNs, condensed mesh-like structures comprising chondroitin sulfate proteoglycans, hyaluronic acid, linking proteins, and tenascin-R, mostly encircle GABA-ergic neurons in various brain regions [31, 32]. The elements of PNNs are dynamic, which play an essential role in synaptic plasticity and protecting recent and remote contextual memory through regulation of GABA release in inhibitory interneurons [32]. The constitution of PNNs gets altered in situations such as fear, reward, and stress [33, 34]. The integrity of the PNN is also noticeably disturbed during trauma, epilepsy, tumor, and schizophrenia [35-42]. Also, digestion or removal of PNNs can interfere with the functional recovery after CNS lesions [43]. The PCM is a basement membrane comprising a sheet-like layer made up of collagen IV, laminins, fibronectin, dystroglycan, and perlecan, which is contiguous with the pia mater of the brain [44]. The ECM consists of proteoglycans, hyaluronic acid, and a small amount of collagens, laminins, and fibronectin.

## Relationship between the ISF, CSF, and the blood-brain barrier

**Figure 2.**
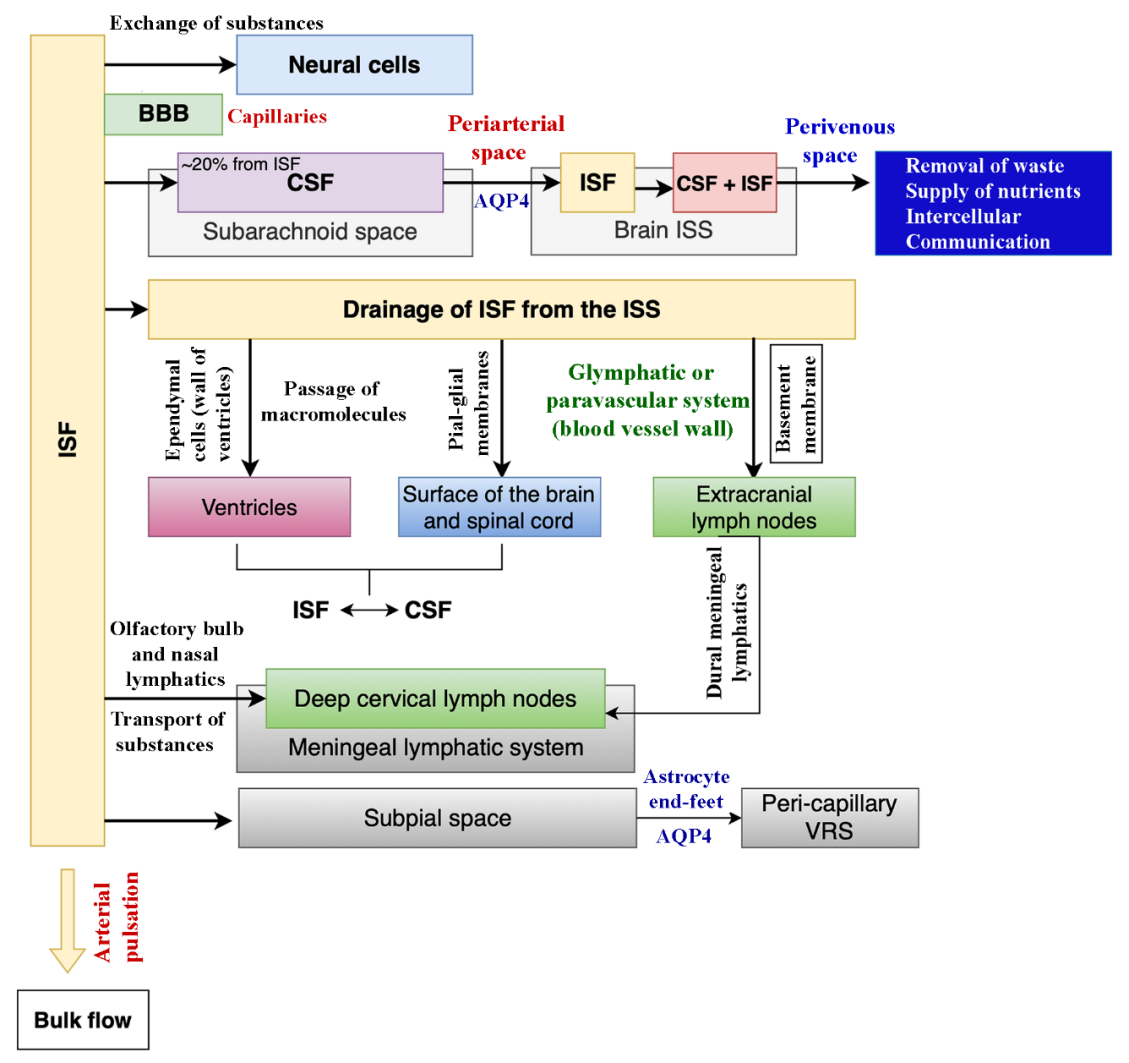
**A schematic showing the various communications of the interstitial fluid (ISF), and the mechanisms by which ISF is drained into the lymphatic system**. ISF interacts with neurons through the capillaries, and the substance exchange between capillaries and neurons is facilitated by the blood-brain barrier (BBB). The cerebrospinal fluid (CSF) resides in the subarachnoid space, and ~20% of the CSF in the brain comes from the ISF. The CSF influx into the brain happens through the periarterial spaces, the convective flow into the ISS occurs through the water channel aquaporin-4 (AQP4, located on astrocyte end-feet) where CSF mixes with the ISF, and then the efflux of ISF occurs through perivenous spaces. The drainage of ISF occurs through three suggested conduits. These include passage through ependymal cells into ventricles, pia-glial membranes into the surface of the brain and spinal cord, and the glymphatic system into extracranial lymph nodes. The ISF is then transported via dural meningeal lymphatics into the deep cervical lymph nodes. Also, the transportation of substances from the ISF into the deep cervical lymph nodes can occur via the olfactory bulb and nasal lymphatics. Furthermore, the end-feet of astrocytes are also involved in the transportation of ISF from the subpial space into the peri-capillary Virchow-Robin space (VRS) through AQP4 channels. The arterial pulsation promotes the ISF bulk flow.

The cerebrospinal fluid (CSF) resides in the subarachnoid space covering the entire surface of the brain and the brain ventricles. The CSF serves as a reservoir for the ISF, and an extensive communiqué between the ISF and CSF helps in the removal of waste products from the brain ISF [1, 45] (Fig. 2). Such an exchange between CSF and ISF is vital for the preservation of homeostasis in the neural microenvironment [46]. As per one estimate, ~20% of CSF in the human brain originates from the brain ISF [1, 47, 48]. A study by Iliff colleagues showed that subarachnoid CSF passes into the brain rapidly, alongside the perivascular spaces adjoining the penetrating arteries and reaches the level of capillaries. Then, aquaporin-4 channels positioned on the perivascular end-feet of astrocytes enable the convective flow of CSF into the ISS. The CSF mixes with the ISF and leaves the brain ISS via perivascular spaces along veins [23] (Fig. 2). A similar process likely also aids the quick entry of nanosized EVs into the brain when administered intranasally. Recent studies have shown that intranasally administered stem cell-derived EVs permeate the entire forebrain and incorporate into different neural cells within 6 hours [49, 50]. Such rapid targeting by EVs is likely due to their quick entry into the subarachnoid space through perineurial spaces around olfactory nerves passing through the cribriform plate and subsequent transportation through CSF flow into the ISF. However, real-time monitoring of EV kinetics *in vivo* using single-photon emission computed tomography (SPECT)/positron emission tomography (SPECT/PET), and magnetic resonance imaging (MRI) will be needed to validate the above possibility.

Some exchange of substances also occurs between the ISF and the cytoplasm of neural cells as the plasma membrane of neurons and glia is selectively permeable to specific ions and molecules [51-55] (Fig. 2). Moreover, the interchange of constituents occurs between the plasma and the ISF through capillaries [56]. Typically, neurons and capillaries are separated by 10-20 µm space, which facilitates a highly efficient diffusion-based substance exchange between capillaries and neurons by a highly selective blood-brain barrier (BBB) [57]. The BBB is composed of capillary endothelial cells, pericytes, and astrocyte end-feet with tight junctions linking the endothelial cells [58, 59] (Fig. 2). The BBB freely allows the passage of water and small hydrophobic molecules such as oxygen, carbon dioxide, and hormones. Besides, the BBB permits the passage of lipid-soluble molecules through simple diffusion, and glucose and amino acids via active transport [60, 61].

## The function of the brain ISS

The ISS of the brain is dynamic, which provides a route for cross-talk between elements of the vascular system and neural cells. The role of ISS includes the facilitation of communication between neural cells, processing and integration of information, and coordinated response to changes in the brain environment [1, 62-66]. Furthermore, the peptides and proteins created in the brain ISS are related to the proteolytic processes involved in cell surface remodeling, protein shedding, and the synthesis of regulatory peptides [67]. It has been suggested that the long-distance passage of such active proteins by ISF-CSF exchange is essential for the onset and maintenance of specific behavioral or motivational states such as fear, appetite, mood, and circadian rhythms [68]. Moreover, the enzymes in the brain ISF degrade and remodel the various ECM proteins secreted by neurons, glia and endothelial cells, which include matrix metallopeptidases (MMPs), and tissue plasminogen activators (tPAs) [69]. Some of these proteins have essential functions. For example, MMPs play a role in learning and memory [70], synaptic plasticity, and repair after brain disorders [71]. tPAs, on the other hand, are involved in neuronal plasticity, ECM degradation, microglial activation, and accumulation of amyloid plaques [72]. Besides, a recent investigation has shown that the brain ISF accumulation of tau, a protein involved in neurodegeneration in Alzheimer’s disease (AD) and tauopathies, is regulated by the sleep-wake cycle [73]. The study also showed that sleep deprivation induces a two-fold increase in the ISF tau, and facilitates tau spreading from the hippocampus into the entorhinal cortex and the locus coeruleus, a region involved in the maintenance of wakefulness [73]. The above observations are consistent with the studies showing the accumulation of beta-amyloid in the healthy brain after a single night of sleep deprivation [74]. Another relevant recent finding is that deep sleep (i.e., during non-rapid eye movement sleep) is associated not only with low-frequency oscillations in neuronal activity and blood oxygenation but also macroscopic changes in CSF flow through the ISS [75]. Such changes in CSF flow can substantially augment ISF volume and removal of metabolic waste products because pulsatile fluid dynamics potentially enhances mixing, diffusion, and clearance of ISF. Because of the lack of lymphatic structures within the brain, the transport and metabolism of substances occur mostly within ECS, which plays a crucial role in many physiological processes such as sleep, memory, and sensory perception. Also, ECS is related to the occurrence and development of major diseases such as AD, Parkinson's disease, and brain tumors. Therefore, it is of immense clinical significance to study the transport of substances within the ECS of the brain.

The ISS also facilitates the passage of neural cell derived EVs into local and distant sites in the brain and to the bloodstream. EVs are broadly classified as microvesicles (MVs) having a size of 100-1000 nm and exosomes (EXs) ranging in size from 30-150 nm. Microvesicles directly bud-off from the plasma membrane of cells [76, 77] whereas, EXs start in endosomes as intraluminal vesicles, leading to the development of multi-vesicular bodies (MVBs) and then secretion of EXs into the ECS via fusion of MVBs with the plasma membrane [78]. EVs are delineated by a phospholipid bilayer and carry nucleic acids and proteins from parental cells [77]. EVs move through the brain ISS and the CSF, contribute to intercellular communication and impact activities such as neurodegeneration, synaptic plasticity, and behavior [79]. Furthermore, studies have shown that information on specific biomarkers in brain disorders can be gleaned by investigating the composition of CNS-derived EVs from the CSF or the blood. It is also possible to analyze the composition of EVs secreted from specific brain cells (e.g., EVs derived from neurons vis-à-vis astrocytes). Indeed, evaluation of the composition of EVs released by neurons and glia into the ISF, CSF, or blood has provided diagnostic and prognostic insights on brain function in many neurological disorders [80-85].

## Drainage of ISF from the brain ISS

The biophysical and biochemical balance between CSF and ISF is crucial for the metabolism of brain cells and drugs, and hence, continuous exchanges between CSF and ISF are critical [1]. Three potential conduits have been suggested for the drainage of ISF from the ISS. One path is via the wall of ventricles through ependymal cells, which allows the passage of macromolecules [10, 46, 86] (Fig. 2). Ependymal cells, covered in a layer of cilia, line the ventricles, and play an essential role in the regulation of CSF production. The second passageway is at the surface of the brain and spinal cord via pia-glial membranes [10, 23, 46, 87] (Fig. 2). A direct exchange between the ISF and CSF at these two conduits have been confirmed through the newly developed tracer-based MRI technique and traditional optical imaging techniques [88]. The other pathway is the blood vessel wall, where ISF flows in the basement membrane directly opposite to the blood flow and reaches the extracranial lymph nodes through the ISF clearance pathway or paravascular system [21, 89-91] (Fig. 2). Studies have revealed lymphatic vessels in the CNS carrying lymph into deep cervical lymph nodes [92]. A widespread web of meningeal lymphatic vessels in the base of the skull makes up the ISS, which facilitates the drainage of metabolic waste products from the ISS of the CNS into the bloodstream. The ISF pathway is mostly active during sleep when the clearance of harmful metabolites such as beta-amyloid increases two-fold relative to the waking state [74]. By using a newly developed in vivo tracer-based MRI technique, Han and his group demonstrated the drainage of ISF from deep brain regions into the surface of the cerebral cortex [10, 46, 93]. In addition, they showed that the traced ISF from the caudate nucleus drained into the ipsilateral cerebral cortex along the myelin fiber tracts and then into the subarachnoid space [10]. A study using an in vivo two-photon imaging also verified the ISF-CSF exchange between the ISS of the cerebral cortex and the CSF in the subarachnoid space [23].

Kiviniemi and associates, using ultra-fast MRI techniques have demonstrated that three discrete pulsation processes in the human glymphatic system facilitate the exchange between ISF and CSF [94]. The swiftest process is the cardiovascular pulsation commencing from basal peri-arterial spaces in the region of the circle of Willis and outspreading centrifugally towards the cerebral cortex. The other two processes include the respiration related pulses dominating in peri-venous spaces in a centripetal fashion and the slow vasomotor wave fluctuations having distinct spatiotemporal patterns [94]. Nakada and Kwee also suggest the involvement of an astrocyte-based system in ISF drainage through water influx into the peri-capillary Virchow-Robin space (VRS). They suggest that the astrocyte system moves the brain ISF from the subpial space into the peri-capillary VRS by the water channel aquaporin-4. They also suggest that ISF flow in the arterial VRS is essential for the clearance of beta-amyloid, whereas the ISF flow in the peri-venous VRS creates a bulk flow of ISF and makes up the necessary volume of CSF together with the CSF produced by the choroid plexus [95]. Another recent study by Teng et al. showed differential effects between the thalamus and the caudate nucleus following aquaporin-4 knockout (KO) in rats. In the thalamus, KO of aquaporin-4 prolonged the half-life of ISF with a decrease in the clearance coefficient but with no changes in the tortuosity of the ISS [96]. In contrast, in the caudate nucleus, KO of aquaporin-4 increased the volume fraction of the ISS and the diffusion coefficient with decreased tortuosity but no changes in brain ISF drainage. The authors attribute this discrepancy in ISF drainage between the two brain regions to the variable distribution of astrocytes and the extent of aquaporin-4 decline [96].

In conditions such as hydrocephalus, increased ventricular pressure causes high ependymal permeability and enhances the flow from CSF to ISF, which leads to interstitial edema of white matter [97]. However, hydrocephalus does not induce edema in the gray matter of the cerebral cortex because of the more efficient drainage from ISF to CSF via pia-glia membranes [2]. A study, using tracer-based MRI, showed that ISF drainage slows down with increased tortuosity of the ISS in the rotenone-induced rat model of Parkinson’s disease. Administration of antiparkinsonian drug madopar partially prevented these changes in ISS and ISF [98]. Another study investigated the effect of olfactory stimulation with eugenol on ISF drainage in the hippocampus and its link with aquaporin 4 [99]. Eugenol significantly increased the activity of hippocampal neurons but reduced the clearance and diffusion rates of Gd-DTPA and A-594 in the hippocampus, implying an interference with the ISF drainage. Eugenol inhalation also slowed down the rate of Gd-DTPA in CSF entering the hippocampus and its clearance. Remarkably, the KO of aquaporin 4 aggravated these processes [99]. Thus, olfactory stimulation can alter the ISF drainage.

## Status of ISS in neurological disorders

The pathological changes in conditions such as glioma or stroke can alter multiple aspects of the ISS, which may include changes in geometry, ECM and its components, the constituents of the ISF, and the transport of substances in the ISS [1, 100, 101]. In glioma, the uncontrolled growth of tumor cells disrupts the standard architecture of brain tissue. Such growth is also accompanied by the degradation of ECM and the active migration of tumor cells [102]. Studies have reported increased volume and/or tortuosity of the ISS in different types of glioma [103, 104]. A recent study using a tracer-based MRI has also shown that implantation of glioma into the thalamus slows-down ISF drainage and alters the direction of ISF drainage [105].

In ischemic stroke, studies have shown decreased ISS volume as a result of cytotoxic edema [106], and increased ISS tortuosity due to a reduction in diffusion caused by dead-space in the microdomains of ISS [107-109]. Also, aquaporin 4 knockout aggravates early brain injury following subarachnoid hemorrhage through impairment of the glymphatic system in the rat brain [110]. In AD, beta-amyloid deposits accumulate in the brain ISS [111-113], which increases the volume and tortuosity of ISS. It is believed that blockage of brain ISF drainage accelerates the abnormal deposition of beta-amyloid in AD, which contributes to both cerebral amyloid angiopathy and neurodegeneration [23, 114, 115]. Changes in the ISS have also been reported in other diseases, such as Parkinson's disease [116], multiple sclerosis [117, 118], and epilepsy [17, 119]. Moreover, another study in a mouse model of AD by Yue and associates suggested that the destruction of beta-amyloid deposits in the ECS using a light-emitting diode with red light facilitated not only the recovery of ISF drainage but also rescued cognitive function [15]. This study suggested a new avenue for treating AD by targeting beta-amyloid in the ISS. Another study suggested that alcohol intake at low-dose levels promotes the clearance of metabolic waste products, including neurodegenerative disease-related proteins through the ISS [120].

## ISS as the route of drug delivery - Available methods and limitations

The failure rate of CNS drugs in both preclinical and clinical studies is much higher than non-CNS drugs [1, 121, 122]. In fact, in conditions such as neurodegenerative diseases, the clinical failure rate for disease-modifying treatments has been 100% [123]. Also, studies have consistently shown limited penetration into the brain tissue when drugs are delivered intravenously. Therefore, it is plausible that the failure of many potentially effective therapeutics for brain disorders is the result of the inability to effectively deliver and sustain adequate drug concentrations within the brain [124]. From this perspective, ISS based drug delivery approaches have considerable interest. One such promising method is called the convection-enhanced delivery (CED), which involves the continuous injection of the therapeutic agent under positive pressure via a catheter implanted into the brain [125-127]. CED is superior to conventional drug delivery approaches as it facilitates the BBB bypass and enhanced ISF drainage with minimal systemic toxicity and better efficacy. CED has been employed for delivering chemotherapeutic agents in several tumor clinical trials and glial cell line-derived neurotrophic factor in Parkinson’s disease [128].

Nonetheless, further refinements are necessary for CED to be translated successfully to the clinic. Notably, a device that avoids the backflow or reflux of injection and mechanical damage to the brain tissue is needed [129-131]. Furthermore, appropriate in vivo monitoring methods are required to measure the concentration and distribution of the injected drug [132-134]. Simple diffusion delivery (SDD) is another local brain drug delivery method that utilizes the concentration gradients to drive pharmaceutical agents to target zones via the brain ISS [135]. Since the brain ISS is divided into different segments based on brain ISF drainage [88, 92, 118, 135], SDD has promise for delivering drugs to specific regions of the brain. However, before targeting drugs to brain regions through an SDD method, the tracer-based MRI technique may need to be used to ensure the brain ISF drainage in the area of the brain where the drug needs to be targeted. One of the significant advantages of SDD is that it can facilitate a full immersion and contact of the drug with target cells, which may allow effective treatment with much smaller doses of the drug [1, 136].

## Conclusions

Many exciting advances occurred in the past decade regarding our understanding of brain ISS. The new developments in the field include identification of differential ISF drainage in distinct functional segments of the brain, and several routes and mechanisms underlying the drainage of ISF into ventricles and the subarachnoid space. Interestingly, tau accumulation in the brain ISS and sleep deprivation augmenting that process have been identified as one of the factors that facilitate the spread of tau in AD. Additional studies have also shown the promise of the removal of beta-amyloid from the brain ISF for improving cognitive function in AD. On the other hand, ISS-based drug delivery, though promising, needs further development and refinement for a successful clinical application of this approach.
